# Supplementary figures and images for: A nonlinear total variation based computed tomography (CT) image reconstruction method using gradient reinforcement
Source: PeerJ. 2024 Jan 8;12:e16715. doi: 10.7717/peerj.16715 (PMC10782945; doi:10.7717/peerj.16715)

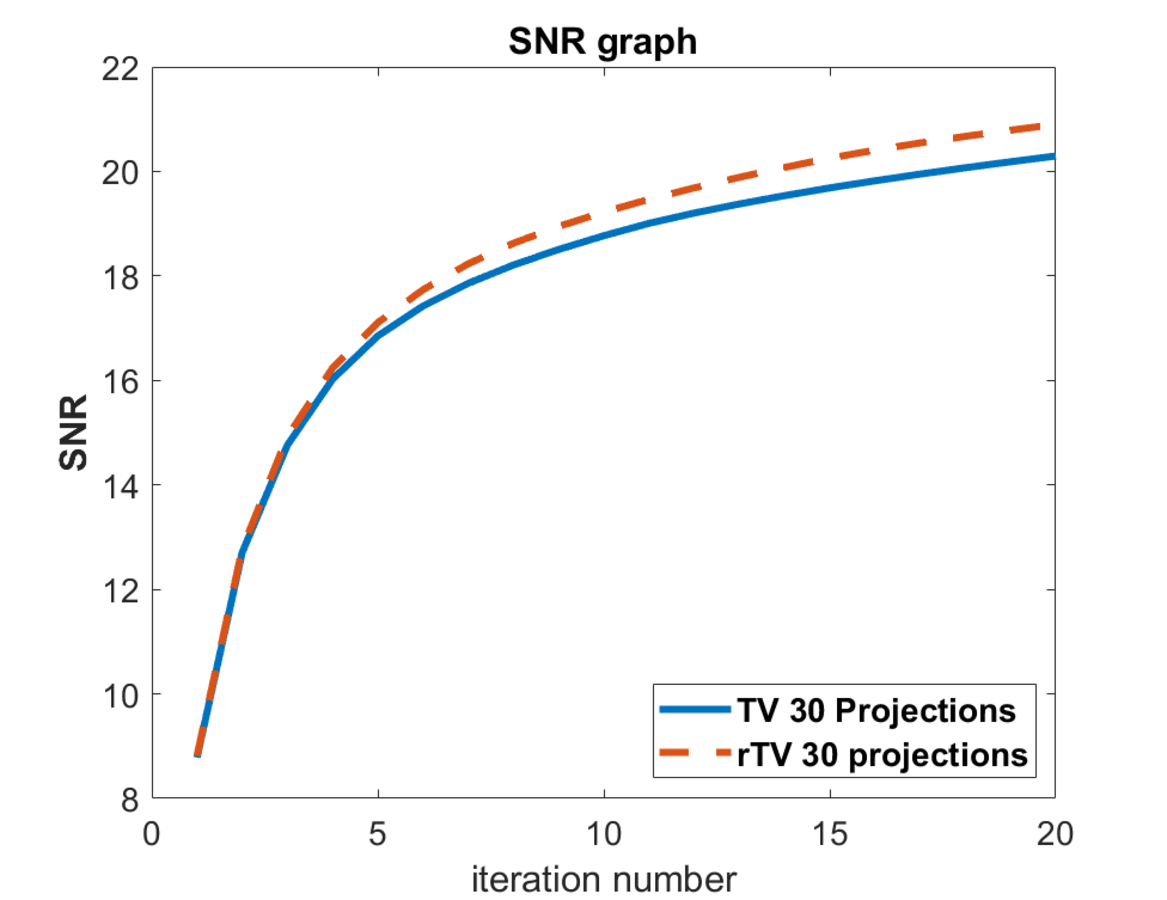

Supplement: Supplemental Information 2 [file peerj-12-16715-s002.png]

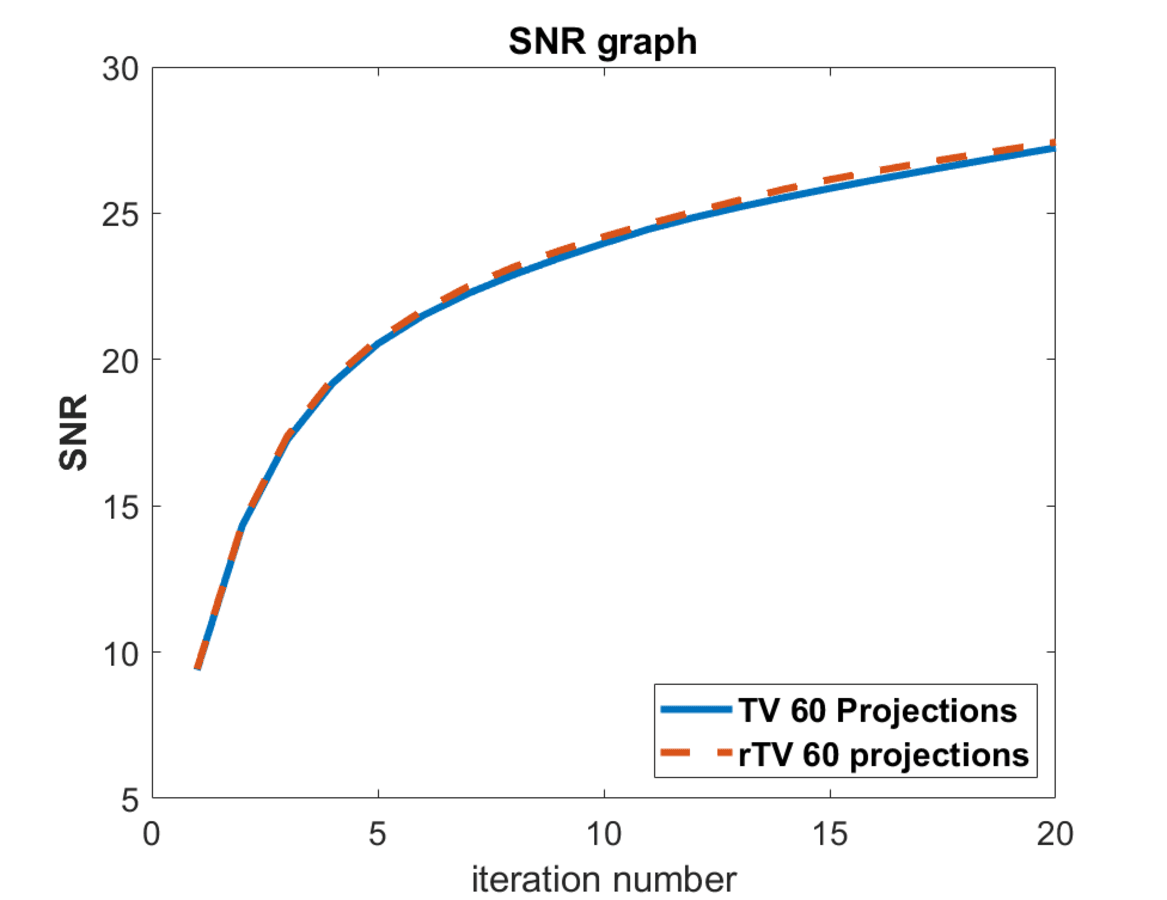

Supplement: Supplemental Information 3 [file peerj-12-16715-s003.png]

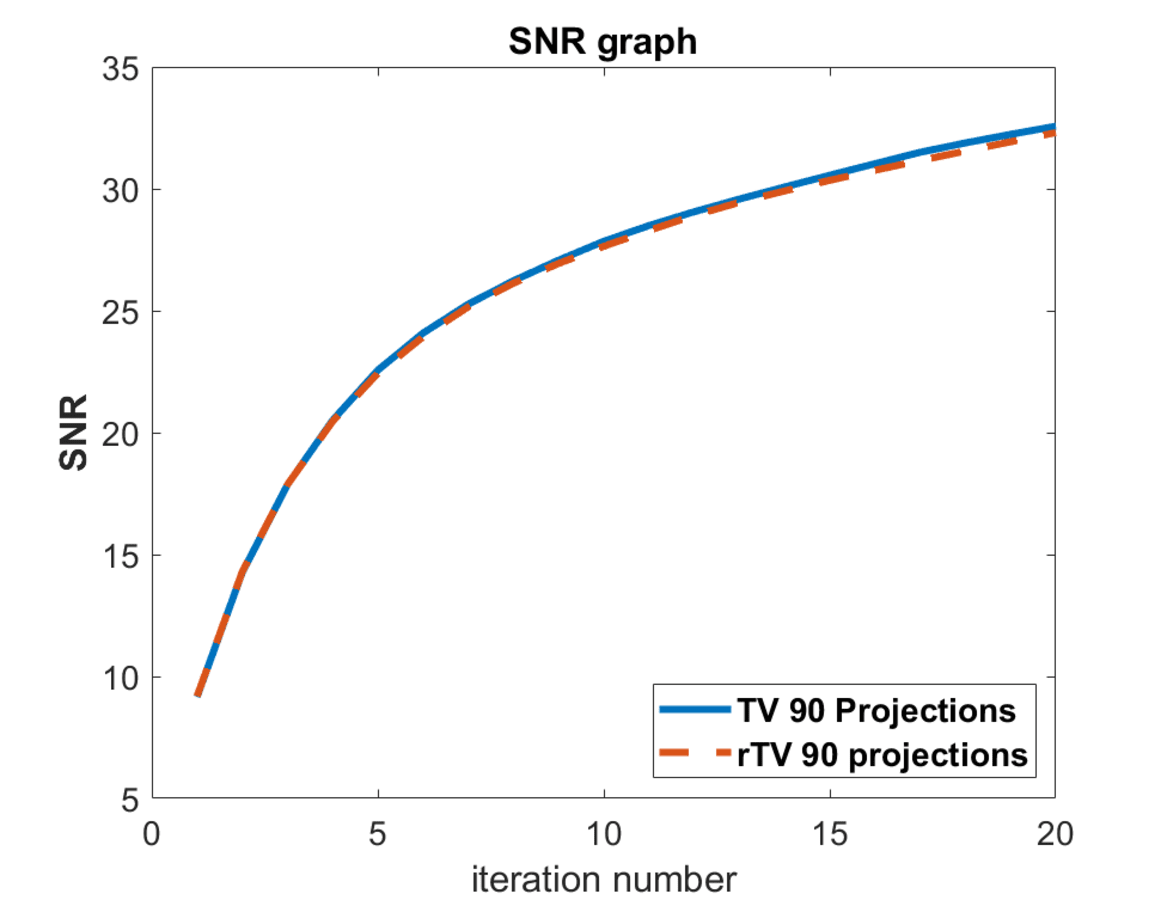

Supplement: Supplemental Information 4 [file peerj-12-16715-s004.png]

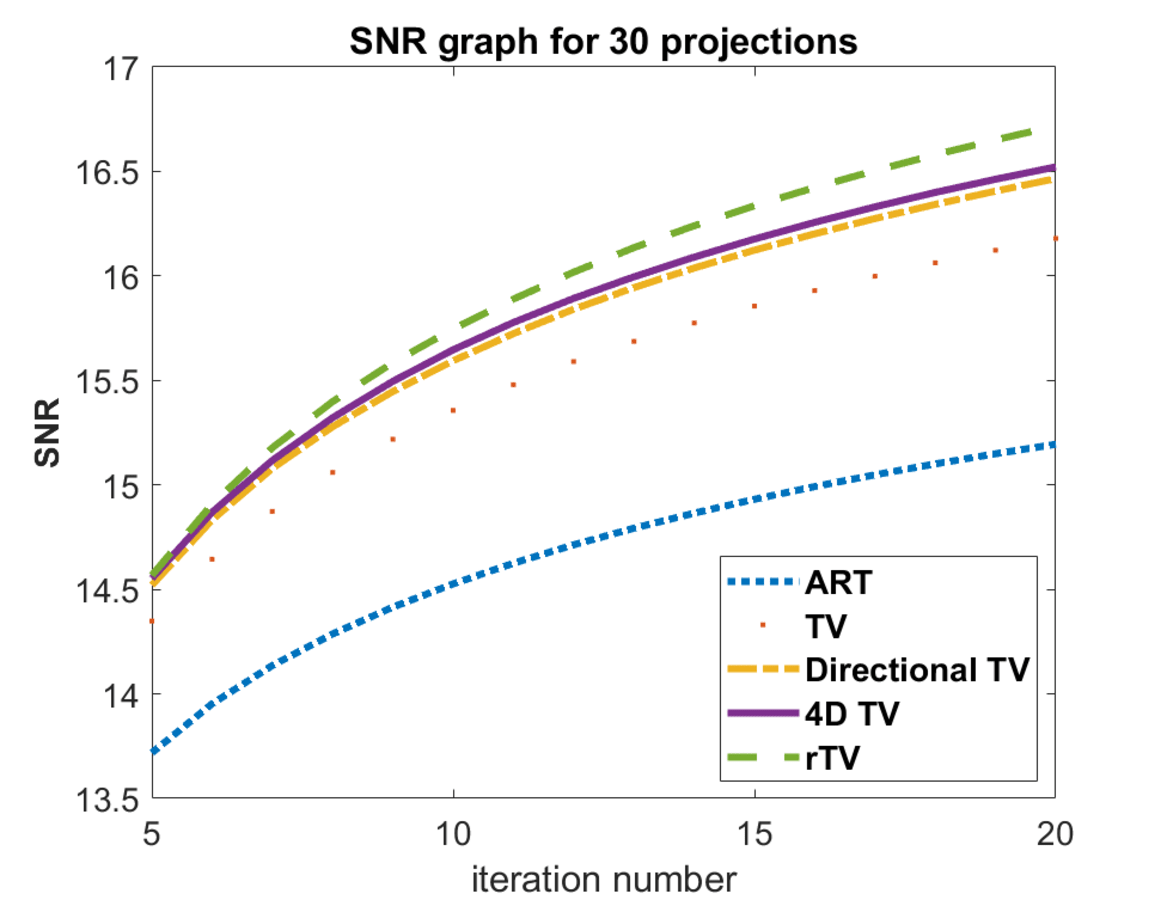

Supplement: Supplemental Information 5 [file peerj-12-16715-s005.png]

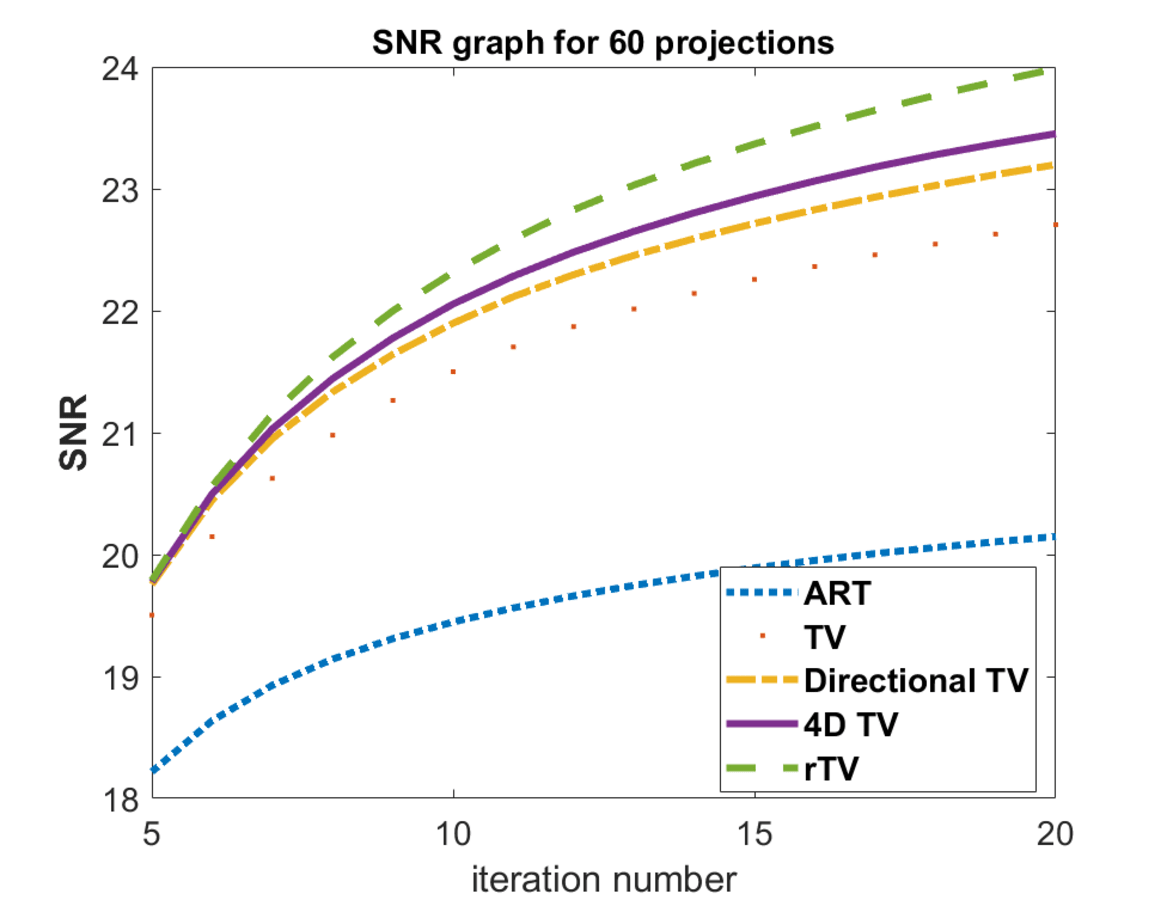

Supplement: Supplemental Information 6 [file peerj-12-16715-s006.png]

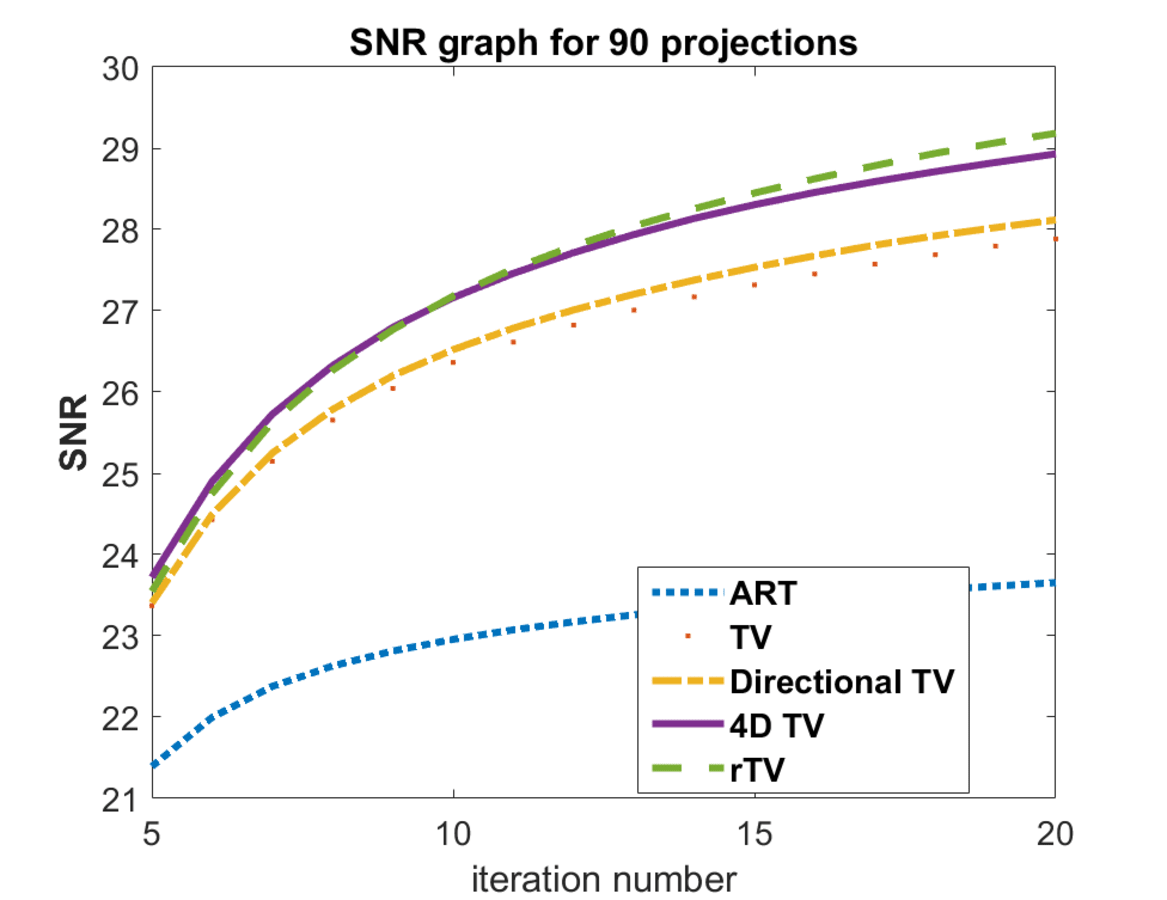

Supplement: Supplemental Information 7 [file peerj-12-16715-s007.png]
